# The history of leishmaniasis

**DOI:** 10.1186/s13071-017-2028-5

**Published:** 2017-02-15

**Authors:** Dietmar Steverding

**Affiliations:** Bob Champion Research & Education Building, Norwich Medical School, University of East Anglia, Norwich Research Park, James Watson Road, Norwich, NR4 7UQ UK

**Keywords:** Leishmaniasis, *Leishmania*, History

## Abstract

In this review article the history of leishmaniasis is discussed regarding the origin of the genus *Leishmania* in the Mesozoic era and its subsequent geographical distribution, initial evidence of the disease in ancient times, first accounts of the infection in the Middle Ages, and the discovery of *Leishmania* parasites as causative agents of leishmaniasis in modern times. With respect to the origin and dispersal of *Leishmania* parasites, the three currently debated hypotheses (Palaearctic, Neotropical and supercontinental origin, respectively) are presented. Ancient documents and paleoparasitological data indicate that leishmaniasis was already widespread in antiquity. Identification of *Leishmania* parasites as etiological agents and sand flies as the transmission vectors of leishmaniasis started at the beginning of the 20^th^ century and the discovery of new *Leishmania* and sand fly species continued well into the 21^st^ century. Lately, the Syrian civil war and refugee crises have shown that leishmaniasis epidemics can happen any time in conflict areas and neighbouring regions where the disease was previously endemic.

## Background

Leishmaniasis is a vector-borne disease caused by flagellated protozoans of the genus *Leishmania*. The disease is widespread in the tropical and subtropical areas and found in 98 countries in Europe, Africa, Asia and America [1]. However, over 90% of new cases occur in just 13 countries (Afghanistan, Algeria, Bangladesh, Bolivia, Brazil, Columbia, Ethiopia, India, Iran, Peru, South Sudan, Sudan and Syria) [2]. It is estimated that between 0.9 and 1.7 million people are newly infected every year, but only a small fraction of them will develop the disease and 20,000–30,000 will eventually die [2].

*Leishmania* parasites are transmitted by the bite of infected phlebotomine sand flies and 98 species of the genera *Phlebotomus* and *Lutzomyia* have been described as proven or suspected vectors for human leishmaniasis [3]. Only female sand flies attack mammals to take blood meals required for the completion of egg development. Some sand flies have a wide host range including canids, rodents, marsupials and hyraxes while others are mainly feeding on humans. Accordingly, human leishmaniasis can have zoonotic or anthroponotic transmission patterns.

In their mammalian host, *Leishmania* parasites live and multiply intracellularly in phagocytic cells within so-called phagolysosomes. Currently, there are 18 different *Leishmania* species described that are pathogenic for humans (Table 1) [4–6]. Although the different *Leishmania* species are morphologically very similar, they cause two main clinical forms, cutaneous leishmaniasis (CL)^1^ and visceral leishmaniasis (VL)^2^, depending on which types of phagocytic cells are invaded. In CL, the parasites infect macrophages resident in the skin. When the host cell is full of parasites, it bursts and the released amastigotes will infect neighbouring macrophages. In VL, however, the released amastigotes are spread by the blood circulation and infect cells of the mononuclear phagocyte system (reticuloendothelial system) of liver, spleen, bone marrow, lymph nodes and the intestine.

**Table 1:** Species of *Leishmania* causing leishmaniasis in humans (adopted and modified according to references [4–6])

Subgenus	Species	Old/New World	Clinical disease	Distribution
*Leishmania*	*L. aethiopica*	OW	LCL, DCL	East Africa (Ethiopia, Kenya)
	*L. amazonensis*	NW	LCL, DCL, MCL	South America (Brazil, Venezuela, Bolivia)
	*L. donovani*	OW	VL, PKDL	Central Africa, South Asia, Middle East, India, China
	*L. infantum* (syn. *L. chagasi*)	OW, NW	VL, CL	Mediterranean countries (North Africa and Europe), Southeast Europe, Middle East, Central Asia, North, Central and South America (Mexico, Venezuela, Brazil, Bolivia)
	*L. major*	OW	CL	North and Central Africa, Middle East, Central Asia
	*L. mexicana* (syn. *L. pifanoi*)	NW	LCL, DCL	USA, Ecuador, Venezuela, Peru
	*L. tropica*	OW	LCL, VL	North and Central Africa, Middle East, Central Asia, India
	*L. venezuelensis*	NW	LCL	Northern South America, Venezuela
	*L. waltoni*	NW	DCL	Dominican Republic
*Viannia*	*L. braziliensis*	NW	LCL, MCL	Western Amazon Basin, South America (Guatemala, Venezuela, Brazil, Bolivia, Peru)
	*L. guyanensis*	NW	LCL, MCL	Northern South America (French Guinea, Suriname, Brazil, Bolivia)
	*L. lainsoni*	NW	LCL	Brazil, Bolivia, Peru
	*L. lindenbergi*	NW	LCL	Brazil
	*L. naiffi*	NW	LCL	Brazil, French Guinea
	*L. panamensis*	NW	LCL, MCL	Central and South America (Panama, Columbia, Venezuela, Brazil)
	*L. peruviana*	NW	LCL, MCL	Peru, Bolivia
	*L. shawi*	NW	LCL	Brazil
*Mundinia*	*L. martiniquensis*	NW, OW	LCL, VL	Martinique, Thailand

*Abbreviations: DCL* diffuse cutaneous leishmaniasis, *LCL* localised cutaneous leishmaniasis, *MCL* mucocutaneous leishmaniasis, *NW* New World, *OW* Old World, *PKDL* post-kala-azar dermal leishmaniasis, *VL* visceral leishmaniasis

The most common form of leishmaniasis is CL with 0.7–1.3 million new cases occurring annually worldwide [2]. CL occurs in three different forms, localised cutaneous leishmaniasis (LCL), diffuse cutaneous leishmaniasis (DCL) and mucocutaneous leishmaniasis (MCL). LCL is characterised by skin lesions and ulcers on exposed parts of the body, leaving permanent scars. DCL is a less common and distinguished from LCL by the development of multiple, slowly progressing nodules without ulceration involving the entire body. MCL is restricted to Latin America. After the initial skin lesion has healed, the disease spreads to the mucous membranes of the nose, mouth and throat. Subsequently, the mucosal ulcers cause destruction of the nasal septum, lips and palate leading to extensive facial disfiguring. VL is the most severe form of leishmaniasis with an estimated 0.2–0.4 million new cases occurring worldwide each year [2]. Without treatment, VL is fatal in over 95% of cases. The symptoms of VL included irregular fever, weight loss, hepatomegaly, splenomegaly (sometimes hepatosplenomegaly) and anaemia.

## Origin of the genus *Leishmania*

### Fossil evidence

The existence of *Leishmania*-like species in prehistorical times is documented in two fossil ambers. The first *Leishmania*-like fossil was found in the proboscis and alimentary tract of a blood-filled female of the extinct sand fly *Palaeomyia burmitis* preserved in a 100 million-year-old Cretaceous Burmese amber [7, 8]. The *Leishmania*-like species was described in a new, collective fossil genus *Paleoleishmania* and named *P. proterus* [8]. Alongside promastigotes and paramastigotes, amastigotes were also found indicating that the sand fly acquired the parasite from blood of a vertebrate during feeding [8]. The presence of amastigotes is suggestive of a digenetic life-cycle of *P. proterus*. The blood cells were subsequently identified as being of a reptile [9]. The second *Leishmania*-like fossil was described as *Paleoleishmania neotropicum* and was found in the extinct sand fly *Lutzomyia adiketis* in a 20–30 million-year-old Dominican amber [10]. Promastigotes, paramastigotes and amastigotes were observed in the gut and proboscis of the sand fly; however, no vertebrate blood cells were found [10]. Nevertheless, the presence of amastigotes and the fact that no monogenetic flagellates colonise sand flies suggest a digenetic life-cycle of *P. neotropicum* with a vertebrate host. This fossil record also provides evidence that Neotropical sand flies were vectors for *Leishmania*-like parasites in the mid-Oligocene to early-Miocene.

### Geographical origin of *Leishmania* species

The genus *Leishmania* has probably evolved in the Mesozoic era (252–66 MYA) prior to the breakup of the supercontinent Pangaea [11]. However, the particular geographical origin of the different *Leishmania* species is a matter of ongoing debate. Three hypotheses are currently discussed.

#### The Palaearctic hypothesis

In 1971, Lysenko [12] suggested that *Leishmania* originated in the Palaearctic region, an area encompassing Europe, Asia north of the Himalayas, northern Arabia and Africa north of the Sahara, in the Palaeocene (66–56 MYA) [13, 14]. This hypothesis is supported by fossil records indicating that ancestral phlebotomine sand flies and murid rodents also evolved in the Palaearctic region during the Palaeocene [15, 16]. Murid rodents were probably important mammalian reservoir hosts as their burrows offered high humidity and shelter from cold for sand flies [13]. Presumably along with its vector and murid host, the parasite spread to the Nearctic region, an area comprising most of North America, including Greenland, Central Florida and the highlands of Mexico, in the Eocene (56–34 MYA) when the Bering land bridge was intact [13]. After the Bering isthmus vanished, *Lutzomyia* sand flies, the vectors of *Leishmania* species in the New World, evolved in the Nearctic during the Oligocene (34–23 MYA) [13]. When the Panama land bridge was formed about 3 million years ago, sigmodontine rodents and *Lutzomyia* sand flies colonised the Neotropical region, an area including South and Central America, the southern Mexican lowlands, the Caribbean islands and southern Florida, in the Pliocene (5.33–2.86 MYA) [12–14, 17]. However, there is evidence that *Leishmania* may have been introduced into the Neotropical region during the Miocene (23–5.33 MYA) before the uplift of the Panama isthmus [11, 14]. Increasing temperature may have been the reason why sand flies began to inhabit the forest canopy with the consequence that arboreal mammals became new hosts for *Leishmania* parasites. Climate change and the adoption of new hosts by the vector may explain the greater diversity of *Leishmania* in the New World compared to the Old World.

#### The Neotropical hypothesis

The speculation that the genus *Leishmania* had originated in the Neotropical region was first suggested by Lainson & Shaw in 1987 [18] and further elaborated by Noyes in 1998 [19]. It was argued that the greater diversity of New World *Leishmania* compared to that of Old World *Leishmania* was evidence for a Neotropical origin of the species [18, 20]. However, the formation of new species may not always appear at a constant rate which would give rise to a larger number of species over longer residence time. In fact, speciation of *Leishmania* in the New World may be attributed to accelerated evolution in the Neotropical region due to climate change, increased host range and geographical isolation. It was suggested that sloths served as the first vertebrate host for *Leishmania* and that during the Eocene the parasite adapted to porcupines [19]. It was further hypothesised that the parasite was introduced into the Nearctic by infected porcupines and into the Palaearctic by an unspecified mammal during the Miocene [19, 21]. However, this hypothesis is incompatible with at least two scientifically established facts. First, fossil records indicate that porcupines did not appear in the Nearctic until the late Pliocene after the Panama isthmus had formed [16, 22], thus about 30–50 million years later than postulated by the hypothesis. Secondly, *Lutzomyia* sand flies, the only vectors of *Leishmania* in the Neotropical, evolved during the Oligocene in the Nearctic and thus about 30 million years too late to serve as insect carrier for the parasite [13].

#### The Supercontinent hypothesis

In 2000, Momen & Cupolilli [23] provided a third hypothesis suggesting that with the breakup of the supercontinent Gondwana in the Mesozoic the subgenera *Leishmania* and *Sauroleishmania* 3 evolved in Africa while the subgenus *Viannia* developed in South America. The subgenus *Leishmania* includes all the Old World species: *L. aethiopica*, *L. donovani*, *L. infantum*, *L. major* and *L. tropica*. As *L. aethiopica* occurs only in Ethiopia and Kenya, it was reasoned that this species originated in Africa [23]. Based on the restricted habitat of the primitive *Arvicanthes-Phlebotomus* system in sub-Saharan Africa, it was presumed that *L. major* most likely also originated on this continent [24]. An East-African origin for *L. donovani* and *L. infantum* has been postulated based on a cladistic analysis of isoenzymes [25]. As humans evolved in East Africa, it was suggested that the anthroponotic transmission of *L. tropica* indicates that this species may also have originated in this part of Africa [23]. In accordance with the first hypothesis it was postulated that the New World species *L. mexicana*, which belongs to the subgenus *Leishmania* and shares many characteristics with *L. major* [18], dispersed into the Nearctic together with its rodent hosts during the Eocene. After entering South America, climatic and ecological factors probably caused further speciation giving rise to *L. venezuelensis*, *L. amazonensis* and *L. waltoni* [5, 23]. *Leishmania chagasi*, another New World species that belongs to the subgenus *Leishmania*, is meanwhile considered to be synonymous with *L. infantum* which was brought to South America in historical times (about 500 years ago by European settlers or their dogs) [26, 27]. With respect to *Leishmania* parasites of the subgenus *Viannia* (*L. braziliensis*, *L. guyanensis*, *L. lainsoni*, *L. lindenbergi*, *L. naffi*, *L. panamensis*, *L. peruviana* and *L. shawi*), which exclusively occur only in the Neotropical, it was hypothesised that these species evolved in South America after the separation of Gondwana [23]. The supercontinent hypothesis reflects much better the available molecular phylogenetic data and was recently corroborated by phylogenomic reconstruction using new bioinformatics methods (SISRS, Site Identification from Short Read Sequences) to identity over 200,000 informative sites across the genome from newly sequenced and publicly available *Leishmania* data [28]. This new study and two recently published analyses also suggest that sloth- and porcupine-infecting *Leishmania*-like trypanosomatids derived from a clade long separated from *Leishmania* species [6, 28, 29]. Consequently, all *Leishmania*-like sloth and porcupine parasites have now been grouped in the genus *Endotrypanum* 4 and in the new genus *Porcisia* 5, respectively [6, 29]. In addition, the worldwide distribution of *L. martiniquensis* supports an ancient global dispersal of the genus *Leishmania* predating the breakup of Gondwana [28]. This suggestion is corroborated by phylogenetic analyses showing that *L. martiniquensis* belongs to the *L. enriettii* 6 complex [30], a clade basal to the clade comprising the subgenera *Leishmania*, *Viannia* and *Sauroleishmania* [6]. Considering the uniqueness of the *L. enriettii* complex, it was proposed to create a new subgenus *Mundinia* for the *L. enriettii* complex that includes *L. martiniquensis* [6].

Based on available data, it can be concluded that leishmanine trypanosomatids originated in mammals in the Mesozoic on the supercontinent Gondwana. Presumably, a monoxenous insect flagellate established itself in mammals and developed into a dixenous species [6, 31]. It is reasonable to assume that with the diversification of mammals, the genera *Endotrypanum*, *Porcisia* and *Leishmania* initially evolved. After the breakup of Gondwana, the genera *Endotrypanum* and *Porcisia* ended up together with their mammalian hosts on the South American continent. During the separation of Gondwana, the genus *Leishmania* was divided and subsequently evolved into the subgenus *Viannia* in South America and into the subgenera *Leishmania*, *Mundinia* and *Sauroleishmania* in Africa. The absence of leishmanial infections in New World lizards and the phylogenetic proximity of the subgenera *Leishmania* and *Sauroleishmania* is probably an indication that *Sauroleishmania* represent a mammalian line that subsequently became adapted to lizards [6, 31]. Finally, in the Eocene, a species of the subgenus *Leishmania* spread from Asia to the Nearctic together with its rodent hosts via the Bering land bridge and evolved into American *L.* (*Leishmania*) species.

### Ancient times

Only a few accounts exit reporting on the occurrence of leishmaniasis in ancient human history. There are descriptions of lesions reminiscent of Oriental sore on tablets in the library of the Assyrian King Ashurbanipal from the 7^th^ century BCE [32]. It is even thought that they were derived from earlier texts dating back to 1500–2500 BCE [32]. A paleoparasitological study of 42 Egyptian mummies from a Middle Kingdom tomb in West Thebes (2050–1650 BCE) found leishmanial mitochondrial DNA in four specimens [33]. Direct sequencing of the amplified DNA fragment revealed that the four mummies were infected with *L. donovani*, suggesting that VL was present in ancient Egypt. Leishmaniasis is also mentioned in the Ebers Papyrus, a collection of ancient Egyptian medical documents dating back to 1500 BCE [34]. This scripture reports a skin condition, known in English as “Nile Pimple”, which supposedly refers to CL. Using immunological analysis, *Leishmania*-infected macrophages were detected in a Peruvian mummy of a 6-year-old girl dated from 800 BCE [35].

Further evidence for the presence of leishmaniasis during antiquity was the knowledge of ancient Arabic societies that individuals with healed Oriental sores were protected from further infections [36]. This insight was used by the people in the Middle East and Central Asia for active immunisation against Oriental sore. They inoculated exudates from active lesions into the buttocks of young children, particular girls or exposed the bottoms of babies to sand flies in order to prevent the development of disfiguring facial scars.

### Middle ages

Arabic scientists were the major chroniclers in the description of CL during medieval times. In 930, the Persian polymath Rhazes (Abū Bakr Muhammad ibn Zakariyyā al-Rāzī, 854–935) described the occurrence of cutaneous sores in the Baghdad region [37]. The first accurate description of Oriental sore was by the great Persian philosopher and physician Avicenna (Abū ʿAlī al-Ḥusayn ibn ʿAbd Allāh ibn Al-Hasan ibn Ali ibn Sīnā, 980–1037). He described a dermal condition known as Balkh sore from northern Afghanistan suggestive of dry skin lesions caused by *L. tropica* [32]. In the New World, disfiguring facial conditions reminiscent of MCL are depicted on Pre-Columbian ceramics since the 5^th^ century [17, 38]. In addition, four female skulls dating back to the 11^th^ century discovered in the archaeological cemetery of Coyo Oriente in the desert of San Pedro de Atacama, northern Chile, provided morphological and molecular evidence of leishmaniasis in South America [39]. The presence of leishmaniasis at high-altitude (note that the Atacama Desert is 2400 m above sea level) where the disease is normally not found, was explained by migration of lowlanders infected with the diseases to the desert highland [39].

### Modern times

#### 16^th^–19^th^ century

From the 16^th^ century onwards, several accounts of skin infections suggestive of Oriental sore were recorded from various places in the Middle East. In many of the reports the conditions described were named according to the place they were acquired and by which they are still known today (e.g. Aleppo boil, Baghdad boil, Jericho boil) [32]. In 1756, the Scottish physician and naturalist Alexander Russell (1715–1768) published a detailed clinical account of both dry and wet forms of Oriental sore when he was practising in Aleppo [40]. He described how the local people distinguished between a ‘male’ and a ‘female’ form of the disease, which most likely correspond to wet zoonotic CL caused by *L. major* and dry anthroponotic CL caused by *L. tropica*, respectively. He provided a detailed description of the development of lesions and mentioned that the diseases heal within 8 months and 1 year. With respect to treatment, he stated “from what I observed, it is infinitely better to apply nothing, than any of the numberless medicines they make use of” but also wrote that he found that a mercurial plaster was most efficacious.

With the Spanish colonisation of the Americas at the beginning of the 16^th^ century, reports appeared by conquistadors and missionaries describing disfiguring facial conditions reminiscent of MCL [39]. One of the first account of MCL was given by the Spanish chronicler Pedro Pizarro (1515–1602) in 1571. He wrote of coca growers working in the lower eastern slopes of the Peruvian Andes who suffered from the destruction of the nose and lips [41].

There are no convincing reports about VL before the 19^th^ century. One of the earliest account of kala-azar was by the military surgeon William Twining (1790–1835) when he published an article in 1827 about patients in Bengal, India, who appeared emaciated with enlarged spleens, acute anaemia and intermittent fever [42]. In 1832, Twining published a book in which he described in more detail the symptoms of kala-azar including the dried-up and scaly appearance of the skin [43]. The first outbreak of kala-azar was already recorded in 1824/25 in the village of Mahomedpore, thirty miles east of Jessore in Lower Bengal, India [44]. From there, the disease spread westwards and reached Burdwan in West Bengal in 1860 [44]. Kala-azar became epidemic and spread to the north of Bengal and to Assam in the following years [44]. The mortality of kala-azar patients in the affected areas was reported to be about 30% [44]. The disease remained endemic in many areas for the next decades. The word kala-azar^7^ was coined in the late 19^th^ century and literally means ‘back disease’. The naming of the disease as kala-azar refers to the greyish discolouration of the skin of light coloured people in the course of the infection.

Although the search for the causative agents responsible for the different forms of leishmaniasis began at the end of the 19^th^ century, it was not before the turn of the century that *Leishmania* parasites were definitively described. However, already in 1885 the Scottish doctor David Douglas Cunningham (1843–1914) saw *Leishmania* parasites in a Delhi boil but did not realised what they were [45]. Subsequently, the Russian army doctor Piotr Fokich Borovsky (Пeтp Фoкич Бopoвcкий) (1863–1932) was the first to recognise that the bodies present in Oriental sore lesions were protozoans [46]. Because he published his findings in an obscure Russian journal in 1898, his observation remained unnoticed.

#### 20^th^ century

**Fig. 1:**
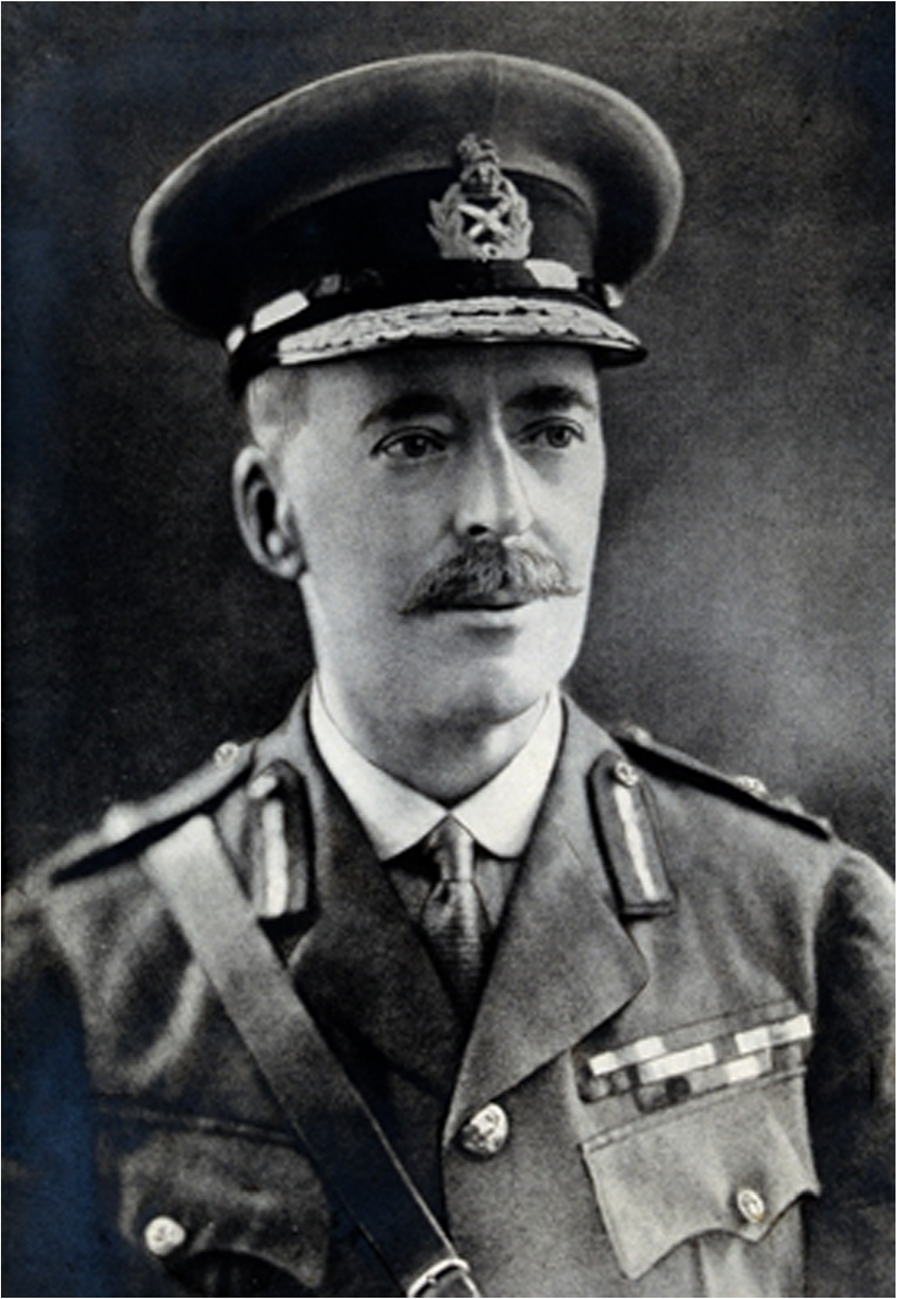
Lieutenant General Sir William Boog Leishman. The genus *Leishmania* was named after the Scottish pathologist who is credited together with Charles Donovan for the discovery of the parasite that caused visceral leishmanioisi (VL). Photo Wellcome Library, London, used according to the Creative Commons Attibution only licence CC BY 4.0

**Fig. 2:**
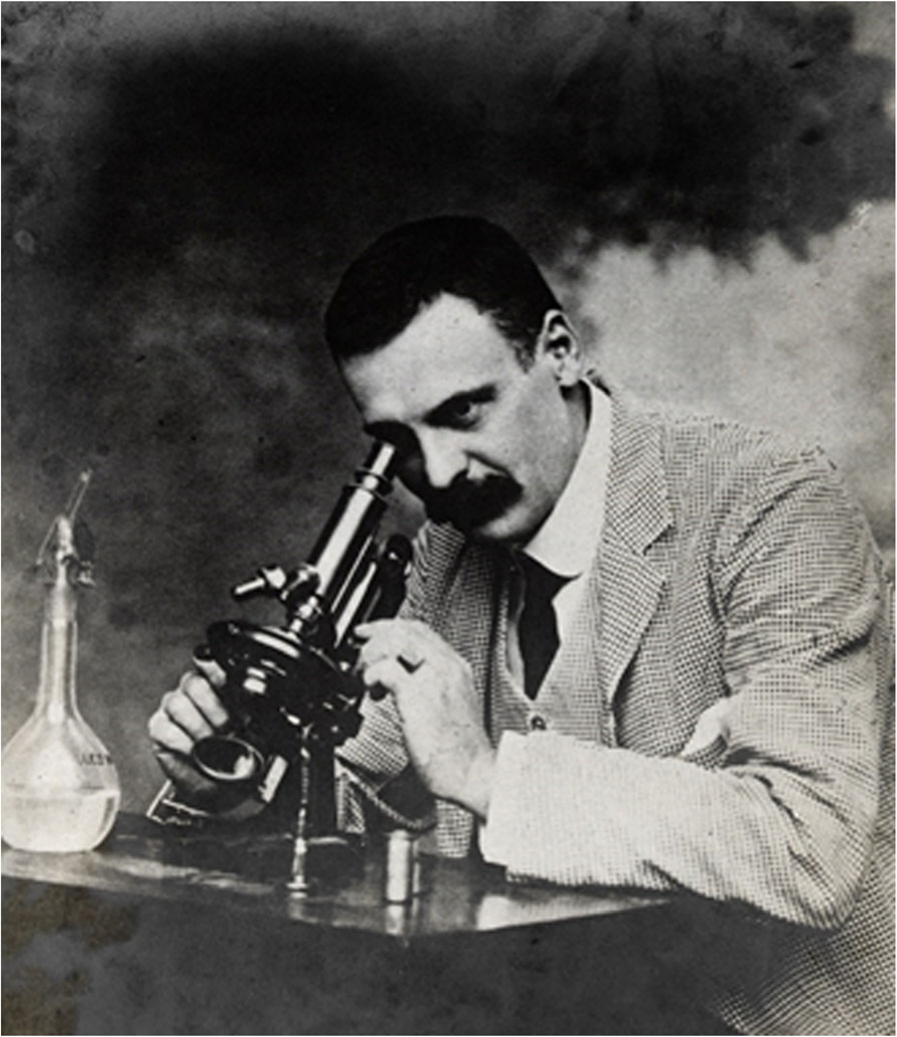
Major Charles Donovan. The species *L. donovani* was named after the Irish doctor who independently of William Leishman discovered the parasite in spleens of patients with kala-azar. Photo Wellcome Library, London, used according to the Creative Commons Attibution only licence CC BY 4.0

In November 1900, the Scottish pathologist William Boog Leishman (1865–1926) (Fig. 1), who served with the British Army in India, discovered ovoid bodies in smears taken *post-mortem* from the spleen of a soldier who died from emaciation and splenomegaly while stationed at Dum Dum, a town near Calcutta [47]. Subsequently, he found similar bodies in an experimentally infected white rat. He published his findings in 1903 and suggested that the ovoid bodies were degenerated forms of trypanosomes and therefore proposed that the illness which he termed ‘Dum-dum fever’ was a form of trypanosomiasis [47]. A few weeks later, the Irish doctor Charles Donovan (1863–1951) (Fig. 2), who was professor of physiology at the Madras Medical College, published a paper reporting that he had found similar bodies in splenic samples taken during life and at autopsy from native Indian subjects with remittent fever and enlarged spleens [48]. As Donovan did not think that the ovoid bodies were degenerated trypanosomes, he sent a slide of the parasite to the French Biologist Félix Étienne Pierre Mesnil (1868–1938) in Paris asking him to show the specimen to his fellow countryman Charles Louis Alphonse Laveran^8^ (1845–1922) who was an authority on protozoan parasites that time. Laveran thought that it was a new parasite of the genus *Piroplasma* [49]. Meanwhile, the British medical doctor Ronald Ross (1857–1932), who was ordered by the Indian government in 1898 to investigate kala-azar, published a paper in November 1903 commenting on the discovery of the ovoid bodies found by Leishman and Donovan in spleen pulp of patients with chronic pyrexia and splenomegaly [50]. He concluded that the ovoid bodies were not degenerated trypanosomes but a novel protozoan organism and that the clinical picture of the cases resembled that of kala-azar. In a follow-up paper, Ross also disagreed with Laveran’s suggestion that the ovoid bodies were parasites of the genus *Piroplama* but that they belonged to a new genus and proposed to name them *Leishmania donovani* [51]. The discussion on the nature of the Leishman’s bodies continued for another year but by the end of 1904 the term *Leishmania donovani* was generally adopted [44]. The related VL causing species *Leishmania infantum* was first described by the French bacteriologist Charles Jules Henry Nicolle (1866–1936) in children in Tunisia suffering from splenic anaemia in 1908 [52]. In the same year, together with his colleague Charles Comte (1869–1943), he also found the parasite in dogs in Tunis [53]. Since then, dogs have been implicated as important reservoir hosts for VL [54].

As already mentioned above, Cunningham and Borovsky were the first who saw leishmanial parasites in Oriental sore lesions but it was the American pathologist James Homer Wright (1869–1928) who was credited for the discovery of *L. tropica*. In 1903, he published a detailed description of the organism from a specimen of a sore of an Armenian girl and named the parasite *Helcosoma* ^i^ 9 *tropicum* [55]. In 1906, the German physician and zoologist Max Lühe (1870–1916) changed the name into *Leishmania tropica* [56]. In 1914, the Russian physicians Wassily Larionovich Yakimoff (Bacилий Лapиoнoвич Якимoв) (1870–1940) and Nathan Isaakovich Schokhor (Haтaн Иcaaкoвич Шoxop) (1887–1941) suggested that *L. tropica* should be divided into the two subspecies *L. tropica minor* and *L. tropica major* based on the size of the parasites found in skin lesions (*L. t. minor*, smaller amastigotes; *L. t. major*, larger amastigotes) [57]. This classification of *L. tropica* became the standard for the next 60 years. Meanwhile, it was discovered that the two subspecies of *L. tropica* were associated with two types of lesions and differences in epidemiology: *L. t. minor* was found to cause dry nodular lesions and to occur in urban environments while *L. t. major* was discovered to produce wet ulcerating lesions and to appear in rural regions [58]. Based on these differences, Bray et al. [59] proposed to classify the two subspecies as *L. tropica* and *L. major*, respectively, in 1973. In the same publication they reported the discovery of a new *Leishmania* species causing a different form of CL in Ethiopia which they named *L. aethiopica* [59].

New World leishmanial parasites were first described independently by the Brazilian doctor Adolpho Carlos Lindenberg (1872–1944) [60] and the Italian physician Antonio Carini (1872–1950) together with his Brazilian colleague Ulysses de Freitas Paranhos (1880–1954) [61] in skin lesions of patients with ‘Baurú ulcers’ from the State of São Paulo, Brasil, in 1909. Two years later, the Italian physician and bacteriologist Alfonso Splendore (1871–1953) found the parasite in mucocutenous lesions of espundia patients [62]. Initially it was thought that the New World parasites were identical with *L. tropica*. In 1911, the Brazilian clinician and scientist Gaspar de Oliveira Vianna (1885–1914) studying leishmanial specimens obtained from a skin lesion of a patient resident in São João de Além Paraiba, Minas Gerais, concluded that the parasite was different from *L. tropica* [63]. He based his decision on apparent morphological differences [41] and named the new species by a *lapsus calami Leishmania brazilienses* [63], which was corrected to *Leishmania braziliensis* by Vianna’s colleague Alfredo Augusto da Matta (1870–1954) in 1916 [64]. Although *L. peruvianna* was already described in 1913, all other New World *Leishmania* species causing LCL and MCL were characterised much later: *L. mexicana* in 1953, *L. guyanensis* in 1954, *L. amazonensis* and *L. panamensis* in 1972, *L. venezuelensis* in 1980, *L. lainsoni* in 1987, *L. naffi* and *L. shawi* in 1989, *L. lindenbergi* in 2002 and *L. waltoni* in 2015 [5, 41]. Another species that previously was associated with leishmaniasis in humans and animals in Colombia and Panama, *L. colombiensis* [65], has been recently reclassified as *Endotrypanum colombiensis* [6].

VL was first recorded in Latin America in the 1930s. Because Aristides Marques da Cunha (1887–1949) and Evandro Serafim Lobo Chagas^10^ (1905–1940) were, for unknown reasons, unable to infect laboratory animals with the parasite from Brazilian cases of VL while that was usually no problem with both *L. donovani* and *L. infantum* causing Old World VL, they thought that they had discovered a new species responsible for VL in the New World and named it *Leishmania chagasi* in 1937 [66]. However, 1 year later, Cunha reported that he succeeded in infecting animals with cultures isolated from cases of American VL and thus concluded that the agent of VL in Latin America is identical to *L. infantum* [67]. More recently, this notion has been supported by modern molecular analysis techniques showing that *L. chagasi* strains could not be distinguished from *L. infantum* strains [68].

The species *L. martiniquensis* was only recently discovered. It was first isolated in 1995, its taxonomical position established in 2002 and named in 2014 [69]. Since 2009, the name ‘*L. siamensis*’ popped up repeatedly in the literature. This ‘new’ species has been associated with leishmaniasis in horses and cattle in Europe and the USA [70–72], and with VL in humans in Thailand [73, 74]. However, as this species has not been properly characterised and described, the name ‘*L. siamensis*’ should not been used [6]. In addition, recent DNA sequence analysis showed that most parasite isolates previously identified as ‘*L. siamensis*’ were identical with *L. martiniquensis* [75]. Thus, ‘*L. siamensis*’ should be regarded as a synonym of *L. martiniquensis* [6].

Although sand flies were suspected early on to be the vectors for transmission of *Leishmania* parasites, it was not until 1921 that this was proven when the French brothers and biologists Edmond Sergent (1876–1969) and Étienne Sergent (1878–1948) demonstrated that scarifying a suspension of ground sand flies into the skin of volunteers resulted in the development of typical Oriental sore lesions [76]. However, the result of this experiment was not generally accepted as proof that sand flies are the vectors of Oriental sore. The actual mode of transmission through the bite of the sand fly was finally demonstrated by the British-Israeli parasitologist Saul Adler (1895–1966) in 1941 when he successfully infected five volunteers with sand flies experimentally infected with *L. tropica* in the laboratory [77]. One year later, it was also conclusively proven that sand flies are the vector of kala-azar [78]. In 1922, the Brazilian doctor Henrique de Beaurepaire Rohan Aragão (1879–1956) showed that sand flies are responsible for the transmission of leishmaniasis in South America [79]. Later it was found that the sand flies transmitting leishmaniasis in the New World belong to the genus *Lutzomyia*. Meanwhile 42 *Phlebotomus* species and 56 *Lutzomyia* species have been implicated in the transmission of leishmaniasis in the Old and New World, respectively [3].

#### Current situation

Leishmaniasis still remains a major health problem in many endemic countries. The total number of annually reported VL cases in the 14 VL high-burden countries (Brazil, China, Ethiopia, Georgia, India, Kenya, Nepal, Paraguay, Somalia, South Sudan, Spain, Sudan and Uganda) has fallen from 60,000 in 2006 to 30,000 in 2014 [80]. This drop in numbers is mainly due to a 5-fold decline in VL cases in India [80]. On the other hand, the total number of yearly reported CL cases in the 12 CL high-burden countries (Afghanistan, Algeria, Brazil, Colombia, Iran, Morocco, Pakistan, Peru, Saudi Arabia, Syria, Tunisia and Turkey) remained unchanged at the high level of about 150,000 over the same period [80].

The increase in the number of leishmaniasis cases observed during the last 25 years throughout the world is due to several factors. Globalisation and climate change are two factors that contribute to the spread of leishmaniasis to non-endemic areas [81]. For example, over the last decades, the number of cases of leishmaniasis in international travellers (tourists and businesspeople) has increased [82]. In addition, the international traffic of blood products has resulted in *Leishmania* infections of patients who never travelled to leishmaniasis endemic regions [81]. The problem here is that no blood bank screens blood preservations for the presence of anti-leishmanial antibodies. There is also evidence that global warming will lead to an extension of the distribution of sand flies more northwards which could result in the transmission of leishmaniasis in hitherto non-endemic regions in the future [81, 83].

Other risk factors for the emergence and spread of leishmanaisis are war and unrest [81]. Currently, of great concern is the outbreak of Old World CL in the Middle East and North Africa. This CL epidemic was triggered by the Syrian civil war and refugee crisis and now affects hundreds of thousands of people living in refugee camps or caught in conflict zones [84, 85]. Before the outbreak of the civil war, the annual incidence of Old World CL in Syria was estimated to be around 23,000 cases [84]. This number has now more than doubled: 53,000 and 41,000 cases were reported in 2012 and in the first half of 2013, respectively [84]. A similar crisis seems to be unfolding in eastern Libya and in Yemen [84]. In addition, outbreaks of leishmaniasis have been recorded from refugee camps in Turkey, Lebanon, Jordan and Tunisia and may soon be reported from Saudi Arabia due to refugee fleeing the current Yemini conflict [84–86].

## Conclusions

From the history of leishmaniasis it is clear that the evolution of the disease is intrinsically tied with human activity. Although the disease probably already affected early hominids, leishmaniasis was not a selection factor in the evolution of humans as was, for example, African trypanosomiasis [87]. Nevertheless, leishmaniasis was spread throughout the world by man during early human migration. In addition, domesticated dogs, one of the main reservoir hosts for VL, seem to have played an important role in the early epidemiology of the disease [88]. The more recent history of leishmaniasis has shown that new *Leishmania* species pathogenic for humans are still to be discovered. The emergence of new forms of leishmaniasis is probably linked to human activity at the edge of or within woodlands. This brings people in closer contact with sand flies that usually feed on wild animals which increases the risk that previously undetected *Leishmania* species may be transmitted to humans. In fact, deforestation and penetration of forests by humans can lead to the adaptation of sand flies to feed on people and their domestic animals near human dwellings and settlements [89]. In many endemic regions, leishmaniasis is an epidemiologically unstable disease that shows a tendency for unpredictable fluctuations in the number of cases. The reasons for this are probably manifold but cultural, environmental and socio-economic factors play an important role. The recent outbreak of CL in conflict zones of the Middle East indicates that war, ecological disasters and forced migration are other factors that are associated with leishmaniasis epidemics.

## Abbreviations

BCE: before common era
CL: cutaneous leishmaniasis
DCL: diffuse cutaneous leishmaniasis
LCL: localised cutaneous leishmaniasis
MCL: mucocutaneous leishmaniasis
MYA: million years ago
VL: visceral leishmaniasis

## Footnotes

1 In the Old World, CL is known as Oriental sore, Aleppo boil, Jeriho boil, Baghdad boil, Balkh sore, Penjdeh sore, Briska button (clou de Briska), Bouton de Crete and Bouton D’Orient. In the New World, the disease is known as Uta, Espundia, Chiclero’e ulcer, Pain bois and forest yaws

2 Visceral leishmaniasis is also known as kala-azar, black fever and Dumdum fever

3 The subgenus *Sauroleishmania* includes all reptile-infecting *Leishmania* species

4 Parasites of the genus *Endotrypanum* are restricted to Neotropical tree sloths and infect erythrocytes of their mammalian host

5 The genus *Porcisia* includes the Neotropical porcupines parasites originally describes as *L. hertigi* and *L. deanei*

6
*Leishmania enriettii* is a leishmanial parasite exclusively found in guinea pigs

7 The word kala-azar was derived from the Hindi/Urdu word for black (kala) and the Persian word for disease (azar)

8 Laveran won the Nobel Prize for Physiology or Medicine in 1907 for his discovery of protozoan parasites as causative agents for infectious diseases

9 For the generic name Homer Wright used the Greek word for ulcer, έλκος

10 Evandro Chagas was the eldest son of Carlos Chagas, who is renowned for the discovery of the causative agent of American trypanosomiasis or Chagas disease, *Trypanosoma cruzi*. Tragically, Evandro Chagas died in an air crash on the 8^th^ of November 1940, aged 35
